# Rare-earth (Gd^3+^,Yb^3+^/Tm^3+^, Eu^3+^) co-doped hydroxyapatite as magnetic, up-conversion and down-conversion materials for multimodal imaging

**DOI:** 10.1038/s41598-019-52885-0

**Published:** 2019-11-08

**Authors:** Nenad L. Ignjatović, Lidija Mančić, Marina Vuković, Zoran Stojanović, Marko G. Nikolić, Srečo Škapin, Sonja Jovanović, Ljiljana Veselinović, Vuk Uskoković, Snežana Lazić, Smilja Marković, Miloš M. Lazarević, Dragan P. Uskoković

**Affiliations:** 1 Institute of Technical Sciences of the Serbian Academy of Science and Arts, Knez Mihailova 35/IV, P.O. Box 377, 11000 Belgrade, Serbia; 2 University of Belgrade, Innovation Center of Faculty of Chemistry, Studentski trg 12-16, 11000 Belgrade, Serbia; 3 University of Belgrade, Institute of Physics, Photonic Center, Zemun, Belgrade, Serbia; 4 Jožef Stefan Institute, Jamova 39, 1000 Ljubljana, Slovenia; 5 University of Belgrade, Vinča Institute of Nuclear Sciences, PO Box 522, 11001 Belgrade, Serbia; 6 Department of Mechanical and Aerospace Engineering, University of California Irvine, Engineering Gateway 4200, Irvine, 92697 CA USA; 7 Universidad Autónoma de Madrid (UAM), Instituto Universitario de Ciencia de Materiales “Nicolás Cabrera” (INC) and Condensed Matter Physics Center (IFIMAC), Departamento de Física de Materiales, 28049 Madrid, Spain; 8 University of Belgrade, School of Dental Medicine, Rankeova 6, 11000 Belgrade, Serbia

**Keywords:** Biomaterials, Biomedical materials

## Abstract

Taking advantage of the flexibility of the apatite structure, nano- and micro-particles of hydroxyapatite (HAp) were doped with different combinations of rare earth ions (RE^3+^ = Gd, Eu, Yb, Tm) to achieve a synergy among their magnetic and optical properties and to enable their application in preventive medicine, particularly diagnostics based on multimodal imaging. All powders were synthesized through hydrothermal processing at T ≤ 200 °C. An X-ray powder diffraction analysis showed that all powders crystallized in *P*6_3_/*m* space group of the hexagonal crystal structure. The refined unit-cell parameters reflected a decrease in the unit cell volume as a result of the partial substitution of Ca^2+^ with smaller RE^3+^ ions at both cation positions. The FTIR analysis additionally suggested that a synergy may exist solely in the triply doped system, where the lattice symmetry and vibration modes become more coherent than in the singly or doubly doped systems. HAp:RE^3+^ optical characterization revealed a change in the energy band gap and the appearance of a weak blue luminescence (λ_ex_ = 370 nm) due to an increased concentration of defects. The “up”- and the “down”-conversion spectra of HAp:Gd/Yb/Tm and HAp:Gd/Eu powders showed characteristic transitions of Tm^3+^ and Eu^3+^, respectively. Furthermore, in contrast to diamagnetic HAp, all HAp:RE^3+^ powders exhibited paramagnetic behavior. Cell viability tests of HAp:Gd/Yb/Tm and HAp:Gd/Eu powders in human dental pulp stem cell cultures indicated their good biocompatibility.

## Introduction

Due to the similarity with the mineral component of bones, calcium phosphates (CPs) have been widely used in reconstructive surgery^1^. Recently, it has been shown that some hybrid composite CPs could be successfully used as biomarkers and agents in theragnostics^2^. Hydroxyapatite (HAp) has been extensively studied as a bone reconstitution material^3^, either as calcium deficient or doped with cobalt^4,5^, zinc^6^, manganese^7^, copper^8^, silver^9^, etc. In order to obtain better osteoconductive and antimicrobial properties, HAp was co-doped with cerium and strontium^10^. Lanthanides, i.e. Rare Earth (RE) elements are also suitable for Ca^2+^ substitution^11^. Their unique magnetic and optical features, which originates from their *4f*-electronic configuration, make them suitable for the preparation of magnetic resonance (MR) agents and highly sensitive diagnostic bioassays^12,13^. The *f-f* transition is impossible according to the Laporte rule and it becomes partially possible by mixing the *4f*^*n*^ with the opposite parity *4f*^*n-1*^*5d*^1^ configuration or through intramolecular charge transfer^14^. Depending on the transition, i.e. relative spacing of the initial and final energy states of photo-excited carriers, photoluminescence occurs through processes of down-conversion or up-conversion. Down-conversion, also known as “quantum cutting”, is a process in which one high-energy photon is “cut” into two lower-energy photons, while up-conversion is an anti-Stokes nonlinear optical process in which one higher-energy photon is emitted for every two or more absorbed lower-energy photons^15^.

Optical modulation induced by two-wavelength excitation^16–19^ and involving RE ion-doped phosphors^20–22^ has been widely reported in the literature. However, the host lattices used to accommodate the optically active dopants have not only suffered from a lack of bioactivity, but have also often exhibit the diametrically opposite effects of cytotoxicity, the reason for which their potential for use in biomedical applications has been, to say the least, questionable. Such low biocompatibility and/or cytotoxicity issues were reported for many of the traditional host matrices, including RE oxides^23^, oxysulfides^24^, and fluorides^25^. Therefore, although the latter materials have been extensively studied as host materials for optically active ions, studies related to CP doping have appeared recently to tackle the problem of weak biocompatibility of the traditional phosphors. Many of the properties an ideal host matrix should possess^20^ including the high tolerance for luminescent centers, the relatively low phonon energy that minimizes non-radiative relaxations and a solid chemical and thermal stability, are also all satisfied by HAp. In addition, HAp can be fabricated in a fully transparent form so as to allow the comparatively free migration of infrared photons through the lattice^26^.

As far as individual studies on doping CP with luminescent ions are concerned, they will be briefly reviewed here. For instance, it was demonstrated that HAp:Gd^3+^ nanoparticles obtained through hydrothermal processing exhibit a strongly linear dependence of luminescence responses on the radiation dose of gamma rays due to which they could be used in thermoluminescence dosimetry^27^. Similarly, ^159^Gd-doped HAp nanorods synthesized as a carrier for radioisotopes were suitable for the detection and treatment of osteosarcoma using magnetic resonance imaging and production of ^159^Gd-^32^P-HAp caused by neutron activation^28^. The doping of HAp with Gd^3+^ ions yielded paramagnetic properties, with ~3-fold enhancement in the longitudinal relaxivity (r_1_ ~ 12 mM^−1^s^−1^), compared to a commercial Gd complex, making the material suitable for T_1_ weighted MR contrast imaging^29^. On the other hand, the doping of HAp with Eu^3+^ has been performed to elucidate structural changes in the local environment after calcium substitution, and it has been shown that Eu^3+^ doped up to 10 at % occupies selectively Ca1 position at the 4 f site^30^. Simultaneous CP and HAp doping with Eu^3+^ was achieved by treating a codfish bone in an aqueous solution of europium nitrate^31^. The co-doping of HAp:Gd with Pr^3+^ led to the enhanced luminescence response of Gd^3+^ at 313 nm, when excited via the Pr^3+^ *f → d* transition at 222 nm^32^. An efficient energy transfer from ^6^P_J_ of Gd^3+^ to ^5^H_J_ of Eu^3+^ achieved in the HAp:Gd/Eu nanocrystals under 273 nm excitation is successfully used for the *in vivo* imaging of cancer in mice^33^. Multifunctional, co-doped HAp:Gd/Eu nanorods obtained through a microwave-assisted synthesis demonstrated a better contrast during MR imaging and computed tomography (CT), high drug adsorption capacity and sustained drug release^34^. The further improvement of magnetic characteristics was accomplished by co-doping HAp:Gd^3+^ with Fe, due to which the obtained nanoparticles showed a high potential for use as agents in positron emission tomography (PET) and single-photon emission computed tomography (SPECT)^35,36^. Lumino-magnetic, as well as lumino-antimicrobial properties have been obtained by co-doping europium with dysprosium^37^ or strontium^38^ in HAp nanoparticles.

All of the abovementioned works were focused on achieving down-conversion photoluminescence that accompanies high-energy UV excitation. On the other hand, the number of studies related to the synthesis of HAp nanoparticles able to adsorb near infrared (NIR) light is scarce. More precisely, only one study reported a successful synthesis of the HAp:Yb^3+^/Ho^3+^ nanoparticles with up-converting properties utilized for the long-term *in vivo* tracking of their osseointegration capacity in rabbits^39^. Nanocomposites composed of HAp and β-tricalcium phosphate (β-TCP) optically activated with the up-converting Yb^3+^/Er^3+^ pair were synthesized in the form of lobes, pellets, tooth root fillings and surface layers on titanium-based implants with the aim of facilitating dentin re-mineralization and implant integration with the surrounding bone tissue^40^. The synthesis of the single-phase Yb^3+^/Tm^3+^ doped β-TCP^41^ and Yb^3+^/Tm^3+^ doped FAp^42^ that could be used as a contrast agent under NIR excitation was also demonstrated, as well as the preparation of the dextran-grafted upconversion FAp:Yb^3+^/Ho^3+^ nanoparticles exhibiting distinct cell fluorescence^43^.

Considering that the NIR excitation, which falls into the biological tissue transparency window, penetrates tissues more deeply and has reduced phonon scattering compared to the UV light, developing a reproducible synthesis procedure for the optically active HAp nanoparticles, especially the up-converting ones, is an important step towards their clinical use. Furthermore, the coupling of fluorescence with magnetism in a single particle is expected to improve sensibility and enable multimodal bio-imaging. For that purpose, we demonstrate here an in-depth characterization of HAp doped with Gd, Gd/Eu, and Gd/Yb/Tm ions focusing on RE^3+^ accommodation in the HAp lattice, as well as on the resulting changes in the energy band gap (E_g_) and in the magnetic and optical properties of the particles.

## Results and Discussion

### The structural and morphological properties of pure HAp and the HAp:RE^3+^ powders

**Figure 1:**
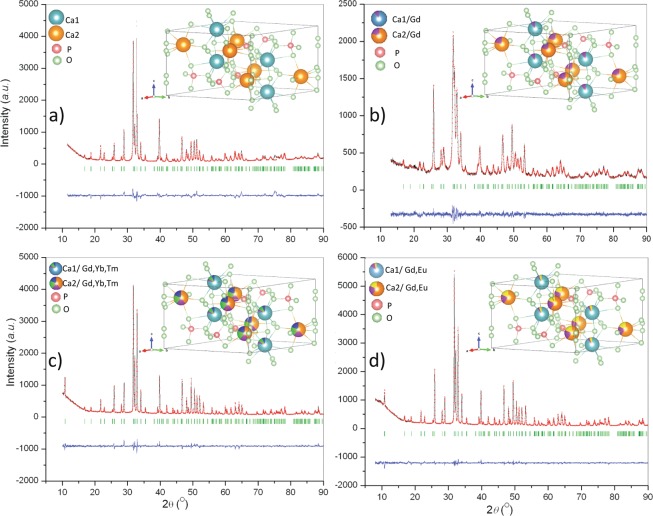
XRPD and Rietveld refinement of (**a**) HAp; (**b**) HAp:Gd; (**c**) HAp:Gd/Yb/Tm and (**d**) HAp:Gd/Eu; XRPD pattern (black), Rietveld refined structure (red) and difference curve (blue); Bragg positions are given as bottom bar lines (green). Visualization of structures of HAp and Re^3+^HAp is shown as inset: Gd-violet, Yb- green, Tm- blue, Eu-yellow.

The Rietveld structural refinement was carried out for all the synthesized powders. The agreement between the observed XRPD patterns and the calculated crystal structures is illustrated in Fig. 1. Since no impurity phases were detected, HAp was refined using the *P*6_3_/*m* space group of hexagonal crystal structure. The refined unit-cell parameters and calculated cell volumes are given in Table 1, while the atomic coordinates and occupancy factors determined for crystallographic Ca1 and Ca2 sites are presented in Table 2. It is apparent that lattice parameters and unit cell volumes decrease continuously as a result of the introduction of smaller RE^3+^ ions to the HAp structure, while the calculated occupancy values for both Ca crystallographic positions clearly show that RE^3+^ ions have a greater affinity for being accommodated at the Ca2 site. This could be a consequence of the higher electronegativity of RE^3+^ ions, resulting in an increased tendency for creating covalent bonds with hydroxyl groups. In the HAp structure, hydroxyl groups are positioned in the channels surrounded by Ca2 cations^44^; therefore, a larger amount of RE^3+^ ions is detected at this site, regardless of their total content. The successful incorporation of smaller RE^3+^ ions at both Ca sites is also reflected in the shortening of the average Ca-O distances (from 2.601 to 2.560 Å and from 2.467 to 2.450 Å for Ca1-O and Ca2-O, respectively, versus the calculated 2.58 Å for the sum of nine-coordinated Ca1^2+^  + O^2−^, and 2.46 Å for the sum of seven-coordinated Ca2^2+^  + O^2−^ ionic radii), Table 3. The calculated P-O bond lengths are in the expected ranges found in other phosphates and their values are 1.532, 1.533, 1.534 and 1.533 for HAp; HAp:Gd; HAp:Gd/Yb/Tm and HAp:Gd/Eu, respectively.

**Table 1:** The refined unit cell parameters and the cell volume of pure HAp and the HAp:RE^3+^ powders.

Sampleas	The unit cell parameters		
	*a* (Å)	*c* (Å)	*V* (Å^3^)
HAp	9.4399(6)	6.8850(4)	531.34(2)
HAp:Gd	9.4303(3)	6.8838(3)	530.16(2)
HAp:Gd/Yb/Tm	9.4248(2)	6.8803(1)	529.28(3)
HAp:Gd/Eu	9.4277(2)	6.8829(1)	529.80(2)

**Table 2:** The refined atomic positions, occupancy factors and atomic displacement in pure HAp and the HAp:RE^3+^ powders.

	Ca1	Ca2	P	O1	O2	O3	O4
HAp		*R*_*f*_ = 3.13	*R*_*b*_ = 3.67				
*x*	2/3	0.2455(7)	0.3975(7)	0.3266(4)	0.5853(1)	0.3390(1)	0
*y*	1/3	0.9930(9)	0.3676(7)	0.4834(2)	0.4649(2)	0.2564(1)	0
*z*	0.0024(1)	1/4	1/4	1/4	1/4	0.0705(1)	0.1912(1)
*B*(Å^2^)	2.82(2)	3.03(1)	3.02(2)	2.87(2)	2.87(2)	2.87(2)	2.87(2)
Occ	1/3	1/2	1/2	1/2	1/2	1	0.1666
HAp:Gd		*R*_*f*_ = 2.99	*R*_*b*_ = 4.46				
*x*	2/3	0.2462(4)	0.3993(1)	0.3294(9)	0.5869(1)	0.3454(3)	0
*y*	1/3	0.9920(5)	0.3692(1)	0.4853(7)	0.4665(8)	0.2614(5)	0
*z*	−0.0003(7)	1/4	1/4	1/4	1/4	0.0677(4)	0.188(2)
*B*(Å^2^)	2.83(16)	2.79(9)	2.06(10)	1.36(10)	1.36(10)	1.36(10)	1.36(10)
Occ (Ca)	0.3247(11)	0.4836(11)	1/2	1/2	1/2	1	0.1666
Occ (Gd)	0.0086(11)	0.0164(11)					
HAp:Gd/Yb/Tm		*R*_*f*_ = 5.15	*R*_*b*_ = 5.66				
*x*	2/3	0.2449(2)	0.3986(1)	0.3285(5)	0.5866(1)	0.3432(4)	0
*y*	1/3	0.9901(3)	0.3660(1)	0.4846(4)	0.4655(5)	0.2584(3)	0
*z*	0.0042(5)	1/4	1/4	1/4	1/4	0.0697(3)	0.1941(13)
*B*(Å^2^)	3.08(10)	3.08(4)	2.59(8)	2.51(9)	2.51(9)	2.51(9)	2.51(9)
Occ (Ca)	0.327(3)	0.481(7)	1/2	1/2	1/2	1	0.1666
Occ (RE^3+^)	0.006(3)	0.0187(7)					
HAp:Gd/Eu		*R*_*f*_ = 2.94	*R*_*b*_ = 2.92				
*x*	2/3	0.2455(2)	0.3989(1)	0.3285(5)	0.5855(1)	0.3393(4)	0
*y*	1/3	0.9915(2)	0.3689(1)	0.4839(4)	0.4664(5)	0.2566(3)	0
*z*	0.0026(4)	1/4	1/4	1/4	1/4	0.0703(3)	0.1947(13)
*B*(Å^2^)	2.77(5)	2.10(4)	2.70(6)	2.33(6)	2.33(6)	2.33(6)	2.33(6)
Occ (Ca)	0.33307	0.49926(5)	1/2	1/2	1/2	1	0.1666
Occ (RE^3+^)	0.00026	0.00074(5)

**Table 3:** The calculated interatomic distances in pure HAp and the HAp:RE^3+^ powders.

HAp	HAp:Gd	HAp:Gd/Yb/Tm	HAp:Gd/Eu
Ca1-O1: 2.389(3) × 3	Ca1-O1: 2.415(3) × 3	Ca1-O1: 2.398(1) × 3	Ca1-O1: 2.402(1) × 3
Ca1-O2: 2.495(7) × 3	Ca1-O2: 2.458(7) × 3	Ca1-O2: 2.487(3) × 3	Ca1-O2: 2.467(3) × 3
Ca1-O3: 2.921(9) × 3	Ca1-O3: 2.793(7) × 3	Ca1-O3: 2.865(4) × 3	Ca1-O3: 2.809(3) × 3
‹Ca1-O› 2.601	‹Ca1-O› 2.552	‹Ca1-O› 2.583	‹Ca1-O› 2.560
Ca2-O1: 2.754(4)	Ca2-O1: 2.707(5)	Ca2-O1: 2.710(2)	Ca2-O1: 2.696(1)
Ca2-O2: 2.382(2)	Ca2-O2: 2.351(7)	Ca2-O2: 2.349(6)	Ca2-O2: 2.364(8)
Ca2-O3: 2.573(9) × 2	Ca2-O3: 2.555(9) × 2	Ca2-O3: 2.319(4) × 2	Ca2-O3: 2.530(3) × 2
Ca2-O3: 2.291(4) × 2	Ca2-O3: 2.328(1) × 2	Ca2-O3: 2.529(9) × 2	Ca2-O3: 2.337) × 2
Ca2-O4: 2.403(9)	Ca2-O4: 2.398(2)	Ca2-O4: 2.399(2)	Ca2-O4: 2.387(8)
‹Ca2-O› 2.467	‹Ca2-O› 2.460	‹Ca2-O› 2.450	‹Ca2-O› 2.454
P-O1: 1.534(5)	P-O1: 1.534(9)	P-O1: 1.534(5)	P-O1: 1.535(3)
P-O2: 1.528(1)	P-O2: 1.533(1)	P-O2: 1.533(2)	P-O2: 1.533(8)
P-O3: 1.533(3) x 2	P-O3: 1.533(2) x 2	P-O3: 1.533(6) x 2	P-O3: 1.534(2) x 2
‹P-O› 1.532	‹P-O› 1.533	‹P-O› 1.533	‹P-O› 1.534

**Figure 2:**
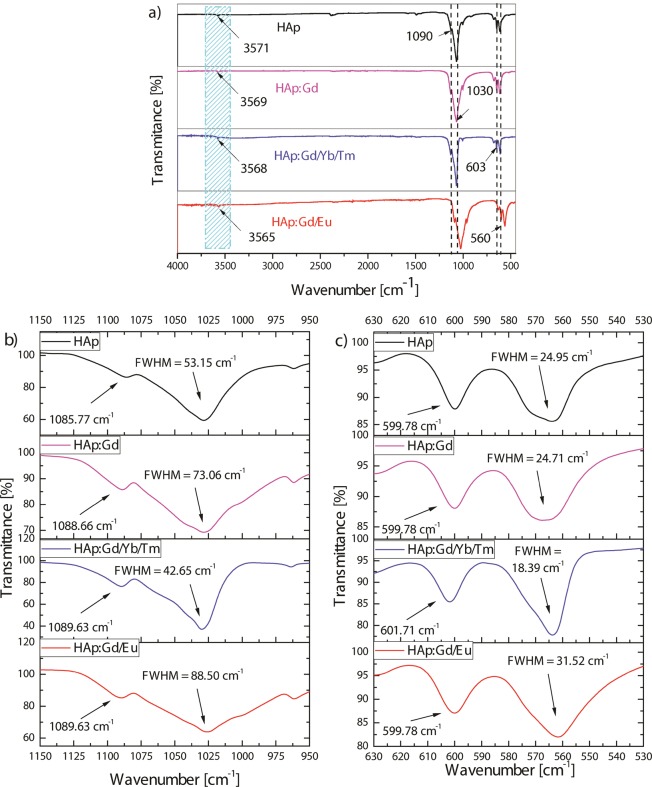
Total FTIR spectra (**a**) and FTIR spectra focusing on v_3_ phosphate stretch in the 950–1150 cm^−1^ wavenumber range (**b**) and FTIR spectra focusing on v_4_ bend in the 530–630 cm^−1^ wavenumber range for HAp and for different HAp:RE^3+^ powders (**c**).

The FTIR spectra of pure HAp and the HAp:RE^3+^ powders, presented in Fig. 2a, exhibit the characteristic absorption bands^45,46^, the most prominent of which are the ν_3_ asymmetric stretching mode of the PO_4_^3−^ group (doublets with maxima at 1028 and 1086 cm^−1^ for pure HAp and the ν_4_ triply degenerated bending mode of PO_4_^3−^ (doublets with maxima at 564 and 600 cm^−1^ for pure HAp). The band observed at 3571 cm^−1^, which originates from the stretching of the structural OH^−^, is also present. The shift to lower values of 3569, 3568 and 3565 cm^−1^ with RE^+^ doping (HAp:Gd, HAp:Gd/Yb/Tm and HAp:Gd/Eu, respectively) indicates slight changes in the structural conformation due to the preferential accommodation of RE^3+^ at the Ca2 position, as it was demonstrated by the Rietveld refinement. As for the characteristic PO_4_^3−^ vibration modes, the prominent ν_3_ band at 1028.40 cm^−1^ did not exhibit any significant shift in RE^3+^-doped powders compared to pure HAp, except for the HAp:Gd/Eu system, for which this band was downshifted by 2.4 cm^−1^. The complementary band of this vibration mode, peaking at 1085.77 cm^−1^ in pure HAp, however, underwent a consistent upshift, to 1088.66 cm^−1^ in HAp:Gd and 1089.63 cm^−1^ for HAp:Gd/Yb/Tm and HAp:Gd/Eu, Fig. 2b. This upshift is indicative of the stiffening of a P-O bond due to doping with RE^3+^, an effect that may have its cause in the somewhat greater degree of disorder in the lattice accommodating these foreign ions compared to pure HAp. In theory, because of the atomic size and charge disparity between Ca^2+^ and RE^3+^, the partial Ca^2+^ → RE^3+^ substitution entails the formation of vacancies on cationic sites, which may lower the cationic coordination of an oxygen within PO_4_^3−^ tetrahedra and stiffen the P-O bond from which this mode originates. This loss of crystalline symmetry is seen from the increase in the full-widths at half-maxima (FWHM) of the major ν_3_ band at 1028.40 cm-1 with RE^3+^ doping. As seen in Fig. 2b, the FWHM increased from 53.15 cm^−1^ in HAp to 73.06 cm^−1^ in HAp:Gd and 88.50 cm^−1^ in HAp:Gd/Eu. However, interestingly, the FWHM of the ν_3_ band at 1028.40 cm^−1^ decreased down to 42.65 cm^−1^ for the material concurrently doped with the largest number of RE^3+^ dopants, namely HAp:Gd/Yb/Tm. The concordant effect is observed for the ν_4_ bending mode of PO_4_^3−^ tetrahedra peaking at 564.10 cm^−1^ in pure HAp, where the FWHM in HAp:Gd/Yb/Tm was lower than that in HAp, in contrast to other RE^3+^-doped systems, for which the FWHM was either equal to that of HAp or higher than it (Fig. 2c). This has suggested that triply doped systems may compensate for the lattice deformation effects of each of the single dopants alone and produce an even higher level of order around the active oscillator than that present in the undoped systems. This effect sheds new light on the prospect of multiply doped systems in HAp (where the number of dopants would be equal to or exceed 3), which have not been explored as intensely as singly and doubly doped ones. As for the shifts in the relatively prominent ν_4_ bending mode of PO_4_^3−^ tetrahedra, no consistent shifts were detected in any of its bands, as in agreement with the lesser sensitivity of bending modes to changes in the dielectric properties of the environment than that of stretching modes.

**Figure 3:**
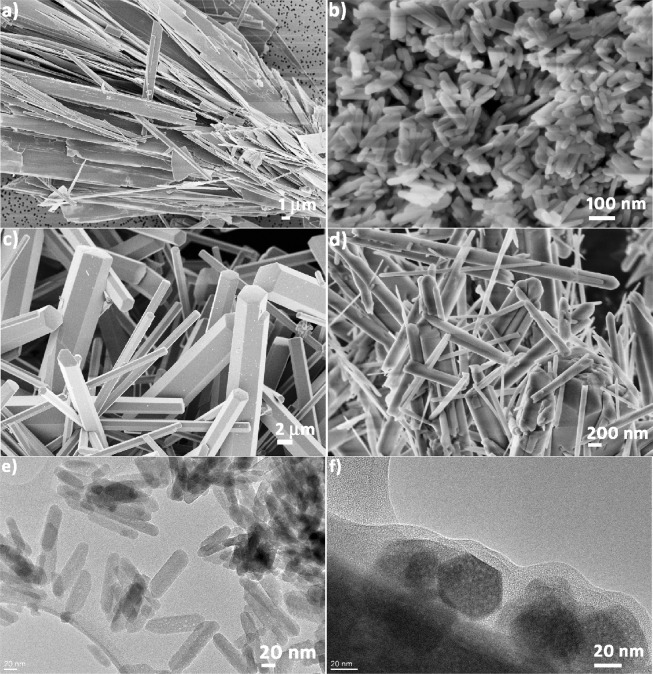
FE-SEM images of (**a**) HAp; (**b**) HAp:Gd; (**c**) HAp:Gd/Yb/Tm; (**d**) HAp:Gd/Eu and TEM images of (**e**) HAp:Gd and (**f**) HAp:Gd/Yb/Tm.

In order to reveal the morphological variations, i.e. the variations in particle size and shape due to RE^3+^ doping, an FE-SEM analysis was performed, Fig. 3a–d. It can be seen that all particles have an elongated shape due to the anisotropic growth of HAp in the *c* axis direction, i.e., preferential adsorption of negatively charged phosphates on the planes with a higher Ca^2+^ exposure (parallel to the *c* axis). In general, plate-like particles with a high length-to-width ratio and a wide size distribution are observed in pure HAp, Fig. 3a. Their size in the longest direction goes beyond 10 μm. Doping with Gd^3+^ inhibits crystal growth and decreases the particle length by up to two orders of magnitude, Fig. 3b. HAp:Gd particles preserve their elongated shape (l = 100 nm) but their length-to-width ratio is much smaller than that of pure HAp, Fig. 3a. The additional thermal treatment of the HAp:Gd/Yb/Tm powder contributes to the growth of well crystallized hexagonal prisms with elongated rectangular sides. The size of the longer prism edge is several dozen micrometers. The jointed parts of the intersecting polyhedra were formed as a result of a mismatched crystals closure. As a result, the petals of hexagonal prisms that share a common base are also detected in this sample, Fig. 3c. The HAp:Gd/Eu sample consists of needle-like particles with somewhat rounder edges. Their length is several micrometers, while their width ranges from 50 to 200 nm, Fig. 3d. Overall, each combination of dopants modifies the particle size and morphology compared to undoped HAp and it is possible that these modifications originate from redistribution of surface charges within the nuclei of growing crystals as the result of the electron charge transfer between the dopants and the lattice cations^47^. The transient electric dipole induced by doping with RE^3+^ ions could affect the diffusion of anions (HPO_4_^2−^, H_2_PO_4_^−^ and OH^−^) from the solution to the growing surface and thus hinder or accelerate growth in particular crystallographic directions^48,49^. It is notable that particle growth occurs through a coalescence mechanism, i.e., the oriented attachment of particles along their longer dimension and subsequent merging. To support the FE-SEM results, a TEM analysis of HAp:Gd and HAp:Gd/Yb/Tm was also performed. While the uniformity of particle sizes and shapes is apparent in the HAp:Gd sample (Fig. 3e), the TEM analysis of HAp:Gd/Yb/Tm reveals that the sample is composed of big and small particles, where both have a well-faceted hexagonal shape (Fig. 3d). Doping with foreign ions can often lead to encapsulation of the major phase by a molten shell of the secondary phase, an effect which minimizes faceting and produces rounder particles^50^. However, one such effect was not evident here upon doping with any of the RE^3+^ ion combinations. Still, by affecting the particle size and shape, doping with RE^3+^ ions is expected to indirectly affect the photoluminescent properties of the material, alongside imparting these properties to it in the first place^51^.

**Figure 4:**
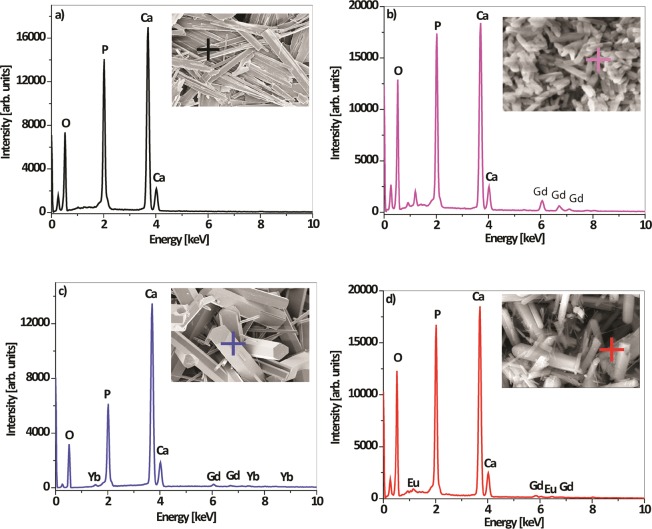
EDS of (**a**) HAp; (**b**) HAp:Gd; (**c**) HAp:Gd/Yb/Tm and (**d**) HAp:Gd/Eu particles.

The EDS analysis at the selected spot provided evidence for the presence of all constituent elements except thulium, the content of which was close to the detection limit (i.e. statistical error). The analyses were repeated in order to check whether the dopants were homogeneously dispersed in different HAp particles. The representative spectra presented in Fig. 4 confirm the purity of all synthesized powders, as well as the presence of calcium (*Kα* at 3.690), phosphorus (*Kα* at 2.013 keV), oxygen (*Kα* at 0.525 keV), gadolinium (*Lα* at 6.056 and *M* at 1.185 keV), ytterbium (*Lα* at 7.414 and *M* at 1.521 keV*)* and europium (*Lα* at 5.845 and M at 1.131 keV) in the labeled particles (the corresponding FE-SEM images are given as insets in Fig. 4).

### The magnetic properties of the HAp:RE^3+^ powders

**Figure 5:**
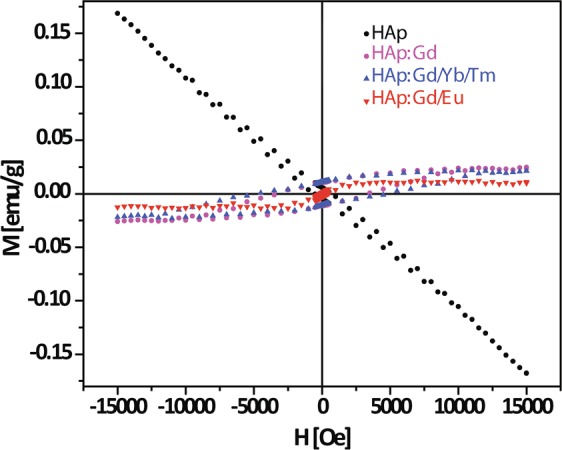
Magnetization curves of the HAp:RE^3+^ powders.

Figure 5 shows the room temperature magnetization curves of HAp and HAp:RE^3+^ powders. Apparently, the presence of dopants alters the magnetic behavior from diamagnetic (pure HAp) to paramagnetic for all HAp:RE^3+^ powders. The shape of the hysteresis loops reflects the existence of a weak long-range magnetic dipole-dipole interaction as a consequence of the incorporation of Gd^3+^ into the HAp matrix. Namely, Gd^3+^ has a high magnetic moment due to the isotropic electronic ground state of ^8^S_7/2_ and the half-filled *f*-orbit and proton relaxation could occur even under low magnetic fields^52^. The coercivity values increase steadily with the increased Gd^3+^ doping concentration in HAp. Values of 0.2, 3.3 and 4.7 kOe were measured for HAp:Gd/Eu, HAp:Gd and HAp:Gd/Yb/Tm powders.

### The indirect E_g_ of HAp and the HAp:RE^3+^ powders

The measured diffusion reflectance spectra of pure HAp and the HAp:RE^3+^ powders were transformed using the Kubelka-Munk function^53^ Eq. (1), where the absorption coefficient α is expressed using the value of diffusive reflectance R_∞_:

1 $$\alpha \approx {(1-{{\rm{R}}}_{\infty })}^{2}/2{{\rm{R}}}_{\infty }={\rm{F}}({{\rm{R}}}_{\infty })$$

Then, the Tauc linearization^54^ Eq. (2), was applied as follows:

2 $${({\rm{\alpha }}{\rm{h}}{\rm{\nu }})}^{{\rm{n}}}={\rm{A}}\ast ({\rm{h}}{\rm{\nu }}-{\rm{Eg}})$$

where h is the Plank constant; ν – the frequency of the vibration, A – the proportional constant and E_g_-the energy band gap. Since hydroxyapatite is considered to have an indirectly allowed transition, the n value is taken to be 0.5^55^.

**Figure 6:**
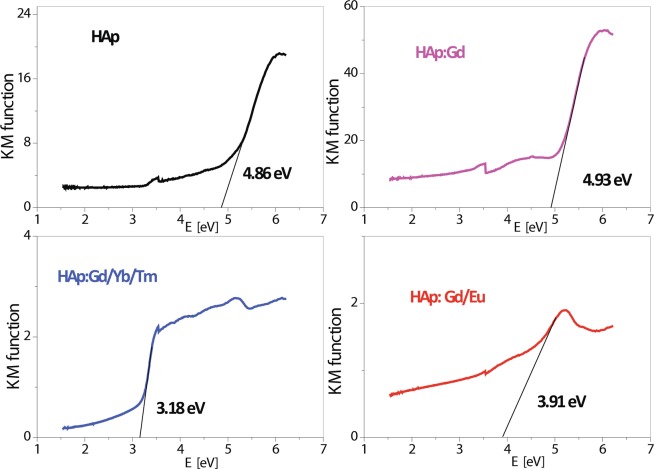
The Tauc plots of diffusion reflectance spectra used to measure the Eg of HAp and HAp:RE^3+^ powders.

According to the literature data, the experimentally detected width of the forbidden zone in the HAp powders is in the 3.9–5.6 eV range, while the theoretically calculated band gap for the HAp monocrystal is 4.51 eV^56^. Here, the energy band gap values of the synthesized samples range from 4.93 to 3.18 eV and decrease in the following order: HAp:Gd > HAp > HAp:Gd/Eu > HAp:Gd/Yb/Tm, see Fig. 6. The shifting of the E_g_ from the initial 4.86 eV in pure HAp to 4.93 eV in the powder where Ca^2+^ was substituted with Gd^3+^ (15 mol%) is associated with increased oxygen vacancies originating from -OH groups, while the additional introduction of RE^3+^ ions obviously changes the type of vacancies created. Based on the computed data, which reveal the nature of defects due to calcium substitution, and the experimental data which correlate the E_g_ shift with the type of vacancies present in the HAp matrix^55,57^, it may be concluded that the introduction of Yb^3+^ and Tm^3+^ as dopants into the position of Ca^2+^ led to oxygen deficiency in the phosphate -PO_4_ groups, while the presence of Eu^3+^ in the HAp matrix induced the creation of mixed vacancies of both an entire -OH group and oxygen from -PO_4_ groups.

### Photoluminescence characteristics

**Figure 7:**
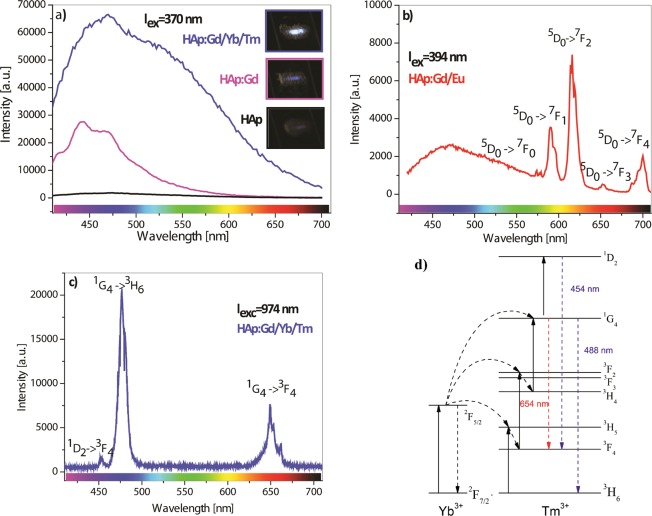
Photoluminescence emission spectra of (**a**) pure HAp, HAp:Gd and the HAp:Gd/Yb/Tm powders under 370 nm excitation; photographs of the powders’ blue emission under excitation; (**b**) HAp:Gd/Eu powder under 394 nm excitation; (**c**) the time-integrated micro-photoluminescence of the HAp:Gd/Yb/Tm powder under 974 nm excitation and (**d**) Energy-level diagram of Yb^3+^ and Tm^3+^ transitions following 974 nm excitation.

All HAp powders exhibit white color under the sunlight regardless of their chemical composition. Under the UV-light irradiation of λ = 370 nm in the dark, the intensity of the emitted pale blue light (inset in Fig. 7a) due to self-activated luminescence follows the same trend as the intensity of the emission spectra shown as inset in Fig. 7a. While the spectrum of the un-doped HAp does not show any luminescence, the introduction of Gd solely and of Gd/Yb/Tm in the HAp crystal structure results in a broadband emission in the measured spectral range comprised of four overlapping components (centered near 440, 470 520 and 575 nm), reflecting the presence of surface and deep-level defects in the HAp structure. The nature of these defects, as indicated by the E_g_ values, is due to oxygen vacancies, which most probably originate from -OH groups from HAp. Namely, when a trivalent RE ions are introduced into the HAp structure, there are two possible explanations for keeping the charge balance: the creation of cationic vacancies or the transformation of the OH ions^−^ to the O^2−^ ions. Taking into account that no other phases were observed in the XRD patterns, the whole amount of the initial calcium must be inside the structure; accordingly, the existence of cationic vacancies can be eliminated as a possibility. Then, the substitution of Ca^2+^ with RE^3+^ must be compensated by the increase of negative charge through OH^−^ to O^2−^ ion transformation^58^.

**Figure 8:**
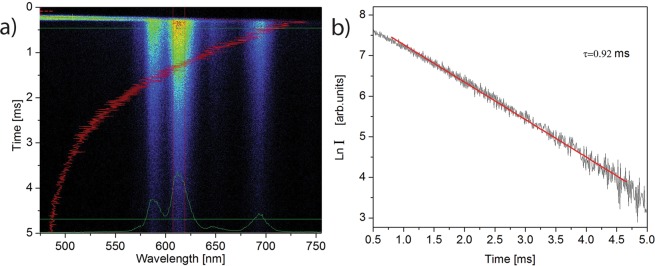
Time resolved emission spectrum (**a**) and fluorescence decay curve of the ^5^D_0_ → ^7^F_2_ emission (**b**) in HAp:Gd/Eu following excitation at 394 nm.

On the other hand, the excitation of co-doped RE emitters (Eu^3+^ and Tm^3+^) leads to the emergence of their narrow emission bandwidths in the PL spectra. Thus, the direct excitation of the Eu^3+^ ion co-doped in the HAp:Gd matrix using λ = 394 nm irradiation results in the emergence of the characteristic bands in the visible spectrum as a result of the Eu^3+^ electron transitions from the first excited metastable ^5^D_0_ level to the crystal field components of the ground ^7^F_0–4_ levels, as indicated in Fig. 7b. It is well known that the intensities and splitting of these bands depend on the local symmetry of the Eu^3+^ ion in the crystal field. If Eu^3+^ occupies an inversion symmetry site in the crystal lattice, the magnetic dipole transition ^5^D_0_–^7^F_1_ is the dominant transition. On the contrary, if Eu^3+^ occupies the non-inversion symmetry site, the electric dipole transition ^5^D_0_–^7^F_2_ dominates in the spectrum. Additionally, in accordance with the (2 J + 1) selection rule for the C_s_ symmetry, the ^5^D_0_ → ^7^F_1_ and ^5^D_0_ → ^7^F_2_ transitions should be split into three and five Stark components, respectively. However, in the case of the C_3_ symmetry, the ^7^F_1_ and ^7^F_2_ levels split into two and three sublevels^59^. Since ^5^D_0_ and 7F_0_ levels are non-degenerate under any symmetry, the number of the emission lines assigned to ^5^D_0_ → ^7^F_0_ transition is equal to the number of the lattice sites occupied by Eu^3+^. As shown by the measured emission spectrum, there are two peaks that correspond to the ^5^D_0_ → ^7^F_0_ transition (at 574 and 579 nm); the maximum of the ^5^D_0_ → ^7^F_1_ transition is positioned at 590 nm with two Stark components (at 590 and 596 nm); the most intense emission due to the ^5^D_0_ → ^7^F_2_ transition at 616 nm is split into three Stark components (at 615, 620 and 626 nm); while the emission due to the ^5^D_0_ → ^7^F_3_ and the ^5^D_0_ → ^7^F_4_ transitions have their maxima at 652 and 670 nm, respectively. This confirms that the Eu^3+^ ions are located at both calcium crystal sites C1 (C_3_ symmetry) and C2 (C_s_ symmetry), as indicated by the Rietveld refinement of XRPD patterns. In addition, a broad emission that comprises two overlapping components (centered at 462 and 532 nm) reflects an increased defect concentration due to doping, as observed in other samples, as well. The luminescence kinetic of HAp:Gd/Eu sample corresponding to the emission from the ^5^D_0→_^7^F_2_ emitting level was obtained at room temperature following excitation at 394 nm, Fig. 8. The lifetime of 0.92 ms is obtained using a single exponential function. Obtained value is slightly higher than reported value of 0.69 ms determined for Eu-doped calcium-deficient hydroxyapatite under excitation at 355 nm^60^ which could be a result of more homogeneous distribution of europium ion in HAp matrix.

Similarly, the excitation of the Yb^3+^ ion, which is co-doped with Tm in the HAp:Gd matrix, leads to the appearance of the characteristic Tm^3+^ transitions in the visible part of spectrum, Fig. 7c, resulting from the synergy of the excited state absorption (ESA) and energy transfer (ET) mechanisms. Beside these two, photon avalanche (PA), cooperative energy transfer (CET), and energy migration-mediated up-conversion (EMU) could also arise, but with lower contribution to up-conversion. The energy-level diagram of these transitions (^1^G_4_ → ^3^F_4_, ^1^D_2_ → ^3^F_4_ and ^1^G_4_ → ^3^H_6_) is presented along with the luminescence spectrum of the HAp:Gd,Yb/Tm sample in Fig. 7d. The first energy transfer step from the Yb^3+^ ion to the Tm^3+^ ion populates the ^3^H_5_ level of Tm^3+^. ^3^H_5_ subsequently decays rapidly to the ^3^F_4_ level, whence it raises to ^3^F_2_ with the second energy transfer step. ^3^F_2_ further decays into ^3^H_4_. The third transfer step raises the Tm^3+^ ion from ^3^H_4_ to ^1^G_4_, which yields the emission at (466–490) nm upon radiative relaxation back to ^3^H_6_. The ^1^G_4_ may relax radiatively to the ^3^F_4_ level, as well, resulting in the emergence of the red emission line at 650 nm. The next energy transfer step from the Yb^3+^ ion to the Tm^3+^ ion populates ^1^D_2_ from the ^1^G_4_ level of the Tm^3+^ ion, yielding the visible blue emission at (453 nm) through back relaxation to the ^3^F_4_ level. The described mechanism is similar to the one observed in the previously synthesized Y_2_O_3_:Yb^3+^, Tm^3+^ nanoparticles^61^ and is good agreement with the one obtained from ytterbium/thulium-doped beta tricalcium phosphate^41^. As it has been pointed out in several studies, the total quenching of “up”-conversion in doped HAp occurs due to the existence of the excitation trapping center on the particle surface (OH acceptors) or due to the distorted symmetry of the luminescent center in the HAp crystal lattice. Our results reveal that the photoluminescence quenching process in particles is partially suppressed due to a more homogeneous distribution of RE^3+^ ions in the HAp matrix (through RE^3+^ ion chelating) and diminished surface quenching centers (through a subsequent thermal treatment of hydrothermally processed powders). Still, the low up-conversion quantum efficiency of 0.01% is calculated for HAp:Gd/Yb/Tm sample. Achieving better emission efficiency is of supreme importance for practical applications of up-converting materials. Despite significant efforts, quantum yield of nanocrystals remains up to be one hundred times lower than that of their bulk equivalents, as evidenced by many reports related to hexagonal NaYF_4_ phase which is currently considered as best matrix for the RE^3+^ ions doping^62^. Apart from the intrinsic parity-forbidden nature of *4 f−4 f* optical transitions, the low quantum yield in HAp arises mainly from the presence of surface quenchers (-OH groups) and lattice defects (oxygen deficiency in the phosphate -PO4 groups), determined by FTIR, which suppress the effective energy transfer through intensification o the nonradiative decay.

**Figure 9:**
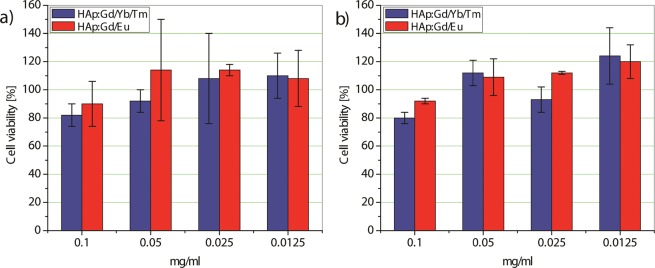
MTT assay comparing the viability of DPSCs incubated with HAp:Gd/Yb/Tm and HAp:Gd/Eu powders for 24 h (**a**) and 72 h (**b**).

The viability of DPSCs after 24 and 72 h exposure to HAp:Gd/Yb/Tm and HAp:Gd/Eu powders at concentrations of 0.1, 0.05, 0.025 and 0.0125 mg/ml, expressed in terms of percentages compared to the surviving cells in the control group is presented at Fig. 9. As it is notable from Fig. 9, the viability of DPSCs was highly preserved after 24 and 72 h exposure, being above 80% for all examined concentrations of HAp:RE^3+^. Insignificant statistical differences of p = 0.027 (24 h) and p = 0.004 (72 h) are detected between DPSCs viabilities in the suspensions with the highest (0.1 mg/ml) and lowest (0.0125 mg/ml) concentrations of HAp:Gd/Eu powders, while statistical difference of p = 0.015 (24 h) is measured for DPSCs viabilities in the suspensions containing 0.1 and 0.05 mg/ml of HAp:Gd/Yb/Er powder. The lack of cytotoxic response to test revealed the safe use of synthesised HAp:RE^3+^ particles for multimodal imaging of dental pulp stem cells.

## Conclusion

HAp nano- and micro-particles were successfully doped with Gd, Gd/Eu and Gd/Yb/Tm in order to obtain magnetic, magnetic-“down”converting and magnetic-“up”converting multifunctional materials. Doping endowed diamagnetic HAp with paramagnetic behavior and affected the particle size and shape, which was still elongated in all cases, coming in the form of plates, rods, needles or hexagonal prisms of different dimensions. The magnetization level and the coercivity of HAp:RE^3+^ increased gradually with the concentration of the Gd^3+^ dopant. The substitution of calcium with RE^3+^ dopants changed both the lattice parameters and the bandgap of the material. The coexistence of emitters at both cationic sites was proven by structural refinement, FTIR and photoluminescence measurements. When excited by the NIR light, HAp:Gd/Yb/Tm particles emitted visible blue light due to the ^1^G_4_,^1^D_2_ → ^3^F_4_ radiative transition. Similarly, HAp:Gd/Eu particles emitted visible red light due to the ^5^D_0_ → ^7^F_0–3_ transitions after excitation with λ = 370 nm. Due to the achieved synergy of magnetism and fluorescence in the HAp:RE particles, it is concluded that they may be a promising multimodal imaging agent, particularly when their optical response is enabled through NIR excitation, which allows for deep tissue imaging without autofluorescence from intrinsic biomolecules. Cell compatibility testing verified the safe use of the synthesized HAp:RE^3+^ particles when administered *in vitro* to dental pulp stem cells isolated from a human patient.

## Materials and Methods

### HAp synthesis – Ca_5_(PO_4_)_3_(OH)

For the synthesis of pure HAp we used a previously developed procedure^63^. First, Solution A (alkaline Ca^2+^ solution) was prepared as follows: 2.8 g of Ca(NO_3_)_2_ × 4H_2_0 (Sigma Aldrich, Germany) was dissolved in 11 ml of distillated water to which 1.8 ml of 25% NH_4_OH (in further text ammonia) was added; then distilled water (further in the text referred to as ‘water’) was filled up to 23 ml in total. Solution B (alkaline PO_4_^3+^ solution) was prepared by diluting 0.53 ml of 85% H_3_PO_4_ with 9.2 ml of water, adding 1.2 ml of ammonia, water up to18.5 ml in total, and subsequently ammonia, once again, up to 30 ml. Solution B was initially preheated to 50 °C in order to dissolve precipitated (NH_4_)_3_PO_4_ crystals and then slowly dripped into magnetically stirred Solution A. The obtained suspension was transferred to a 100 ml Teflon-lined stainless steel autoclave. The residues from the glass beaker were washed out carefully with 17 ml of water, making 70 ml of the reaction suspension in total. Hydrothermal reaction was carried out at 150 °C (6 h). When the reactor was cooled down to room temperature, the precipitate was centrifuged, washed repeatedly until pH 7 and subjected to the two-step lyophilization process (first at T = −10 °C, p = 0.37 mbar, τ = 1 h; and at T = −54 °C, p = 0.1 mbar, τ = 3 h).

### HAp:Gd synthesis – Ca_4.775_Gd_0.15_(PO_4_)_3_(OH)

For doping HAp with 3 at.% of Gd^3+^, an additional solution (C) was prepared by dissolving 0.1407 g of GdCl_3_x6H_2_O (Merck, Germany) in 8 ml of water. The rest of the synthesis procedure was the same, except for the concentration of Solution A (which was modified in accordance with the given stoichiometry) and the fact that Solution C was added dropwise simultaneously with Solution A into the preheated Solution B.

### HAp:Gd/Yb/Tm synthesis – Ca_4.775_Gd_0.03_Yb_0.1_Tm_0.02_(PO_4_)_3_(OH)

To ensure the concurrent doping of HAp with Gd^3+^, Yb^3+^ and Tm^3+^, EDTA–assisted hydrothermal synthesis was applied. For this purpose, the following nitrate solutions were prepared by dissolving 0.0331 g of Gd(NO_3_)_3_ × 6H_2_O, 0.1098 g of Yb(NO_3_)_3_ × 5H_2_O (Merck, Germany) and 0.0218 g of Tm(NO_3_)_3_ × 5H_2_O in 8, 5 and 4 ml of water, respectively. Separately, 3.68 g of EDTA (Sigma, Germany) was dissolved in 9 ml of water at 60 °C. To achieve a transparent solution, 2 ml of ammonia was added. The obtained solutions were mixed together with a magnetic stirrer for 10 min after which Solution A (the concentration of which was modified in accordance with the given stoichiometry) was added to the mix. The common complex of metal chelates was dripped slowly into the preheated Solution B. The obtained suspension was transferred to a 100 ml Teflon-lined stainless steel autoclave and heat treated at 200 °C (11 h). When the reactor was cooled down to room temperature, the precipitate was centrifuged, washed repeatedly until pH = 7, dried and treated thermally for 2 h at 350 °C.

### HAp:Gd/Eu synthesis – Ca_4.91_Gd_0.02_Eu_0.04_(PO_4_)_3_(OH)

For the co-doping of HAp with Gd^3+^ and Eu^3+^, 0.0217 g of Gd(NO_3_)_3_ × 6H_2_O (Merck, Germany) and 0.0411 g of Eu(NO_3_)_3_ × 5H_2_O (Merck, Germany) were dissolved in 8 and 5 ml of water, respectively. The rest of the procedure was as in the case of the HAp:Gd/Yb/Tm synthesis.

### Powder characterization

The crystal structure of the synthesized powders was determined based on an X-ray powder diffraction (XRPD) analysis using a Philips PW 1050 diffractometer with Cu *Kα*_1,2_ (*λ* = 1.54178 Å) radiation. The patterns were collected from 8 to 110° 2*θ*, using a step size of 0.02° and a counting time of 12 s per step. The Rietveld refinement was performed using the *FullProf* software^64,65^. The starting model was based on the unit-cell parameters calculated using the LSUCRI software, while the atomic positions used were reported previously in the literature^66,67^. During the refinement, the Thompson–Cox–Hastings (TCH) pseudo-Voigt peak profile function was used. The occupation parameters were varied for both cation sites while the corresponding isotropic atomic displacement parameters were kept at fixed values. In order to preserve the geometry of the PO_4_ tetrahedron, a geometrical restraint of 1.53 Å was used to determine the P-O bond distance.

Fourier transform infrared spectroscopy (FT-IR) was performed on a Nicolet iS10 FT-IR Spectrometer with a Smart iTR Diamond Attenuated Total Reflectance accessory (Thermo Scientific Instruments) in the spectral range from 400 to 4000 cm^−1^. The morphological features of the particles were investigated by means of both field emission scanning and transmission electron microscopy (Carl Zeiss ULTRA Plus FE-SEM and JEOL JEM 2100 F TEM equipped with a Schottky type field emission source and a cryo pole piece operating at 200 keV) coupled with energy dispersive spectroscopy (EDS). A vibrating-sample magnetometer (VSM, 7397 Lake Shore) was used to detect the magnetic properties of the samples at room temperature in a maximum magnetic field of ±15 kOe.

The photoluminescence (PL) measurements were performed at room temperature in air on the Spex Fluorolog spectroflurometer system with C31034 cooled photomultiplier, utilizing a 500 W Xenon lamp as the excitation source with λ_ex_ = 370 and 394 nm. The time resolved emission of HAp:Gd/Eu was measured using the Optical Parametric Oscillator (Vibrant OPO) as the excitation source tunable over a spectral range from 320 nm to 475 nm. The emission spectrum and luminescence lifetime were analyzed by using Hamamatsu streak camera system equipped with the spectrograph. The time-integrated micro-photoluminescence (µ-PL) analysis of the HAp:Gd,Yb/Tm sample was performed in air at room temperature under continuous-wave optical excitation at 974 nm (Ti:Sapphire 3900 S laser pumped with 532-nm Millenia eV, from Spectra Physics). The laser beam was focused onto 3 µm diameter spot on the sample surface using × 50 microscope objective lens (Nikon, NA = 0.55, WD = 8.7). The emitted light was collected by the same objective lens, dispersed by a single-grating monochromator (Horiba/Jobin Yvon 750 M, spectral resolution of ~350 μeV) and detected with a liquid nitrogen-cooled CCD camera (Symphony II, Horiba Scientific). To block the excitation laser from entering the monochromator, a 800 nm short-pass filter (Melles Griot 03SWP418) and an infrared cut-off glass filter (Newport KG-5) were inserted in the optical collection path. The presented µ-PL spectrum was recorded for the excitation power density of ~8.3 mW/cm^2^, but it is noteworthy that a lower power density of 2 mW/cm^2^ was sufficient to excite the up-conversion process in material. The luminescence quantum yield, defined as the ratio of the number of photons emitted to the number of photons absorbed, is derived upon direct excitation of the sample under study, as well as of the reference silicon substrate covered with a thin high-reflectivity metal (i.e. gold) layer. The estimate takes into account: the numerical aperture of the microscope objective, losses induced by all optical elements in the collection path, as well as the efficiency of the diffraction grating and of the charged couple device detector used in our micro-photoluminescence experimental setup. The diffuse reflectance spectra were recorded on a Perkin Elmer Lambda 35 UV–Vis spectrometer equipped with a diffuse reflectance accessory.

### Biological assays

#### Isolation of dental pulp stem cells (DPSCs)

Semi-impacted wisdom tooth from the patient (21 years old) was used for isolation of DPSCs. Atraumatical tooth extraction was performed at the Clinic for Oral Surgery, School of Dental Medicine, University of Belgrade, Belgrade, Serbia, after having obtained a written informed consent form. The study was approved by the Ethics Committee and Review Board of the School of Dental Medicine, University of Belgrade (Protocol number 36/5). *In vitro* experiments were performed in accordance with relevant regulations and guidelines (EU Directive 2004/23/EC). Tooth was immediately transported to the laboratory and further processed under sterile conditions. Tooth surfaces were thoroughly rinsed with phosphate buffered saline solution (DPBS, Thermo Fisher Scientific, Waltham, MA, USA), and dental tissues were isolated as previously described^68^. Briefly, the dental pulp was extracted with an endodontic instrument, after having exposed the pulp chamber by crushing the tooth with a sterile clamp. Tissue was cut into 1 mm^3^ pieces and transferred into culture medium (Dulbecco’s Modified Eagle Medium (DMEM) supplemented with 10% fetal bovine serum (FBS) and 1% antibiotic-antimycotic solution (all from Thermo Fisher Scientific, Waltham, MA, USA). The cells were maintained at 37 °C in humidified atmosphere containing 5% CO_2_. The culture medium was changed every 2–3 days. Cell cultures were passaged after reaching 80% confluence. The MTT assay was done on sixth-passage cells.

#### MTT assay

For evaluation of cytotoxicity, HAp:Gd/Yb/Tm and HAp:Gd/Eu suspensions in medium were prepared with four different concentrations (0.1, 0.05, 0,025 and 0.0125 mg/ml). For each concentration, adequate mass of powder was aseptically weighted and suspended in medium, shaken vigorously and sonicated for 3 min. DPSCs were seeded in a 96- well plate (5,000 cells per well) and incubated at 37 °C in humidified 5% CO_2_ atmosphere. After 24 h 100 μl of the HAp:Gd/Yb/Tm or HAp:Gd/Eu suspensions were added (0.1, 0.05, 0,025, 0.0125 mg/ml) in each plate. Incubation with the cell cultures was stopped after 24 and 72 h by discarding of spent media. Complete medium containing 3-(4,5-dimethylthiazol-2-yl)-2,5 diphenyltetrazolium bromide (MTT, 0.5 mg/ml) (Sigma-Aldrich, St. Louis, USA) was added to each well and then incubated for additional 4 h, as previously described by Castiglioni^69^. The supernatant was discarded and formazan crystals were dissolved in 100 μl dimethyl sulfoxide (Sigma-Aldrich, St. Louis, USA) by shaking in duration of 15 min at 37 °C. Optical density was measured at 540 nm using ELISA (Enzyme-linked immunosorbent assay) microplate reader (RT-2100c, Rayto, China). Three wells without powders were used as a control group. The experiments were done in triplicate and repeated two times. Cell viability, expressed by the ratio of absorbance of the cells incubated with HAp:Gd/Yb/Tm or HAp:Gd/Eu suspensions to that of the cells incubated with culture medium only, were given in diagram as the mean ± standard deviation.
